# Supplementary material for: Genetic comparisons of interleukin-17 reveal a framework for complex signaling evolution
Source: bioRxiv. 2026 Apr 14:2026.04.13.718218. Preprint. [Version 1] doi: 10.64898/2026.04.13.718218 (PMC13105070; doi:10.64898/2026.04.13.718218)
Supplement: Supplement 3 [file media-3.zip › Supplemental files for paper/ERC/IL17xIL17_comparisons/IL25_IL17RA.pdf]

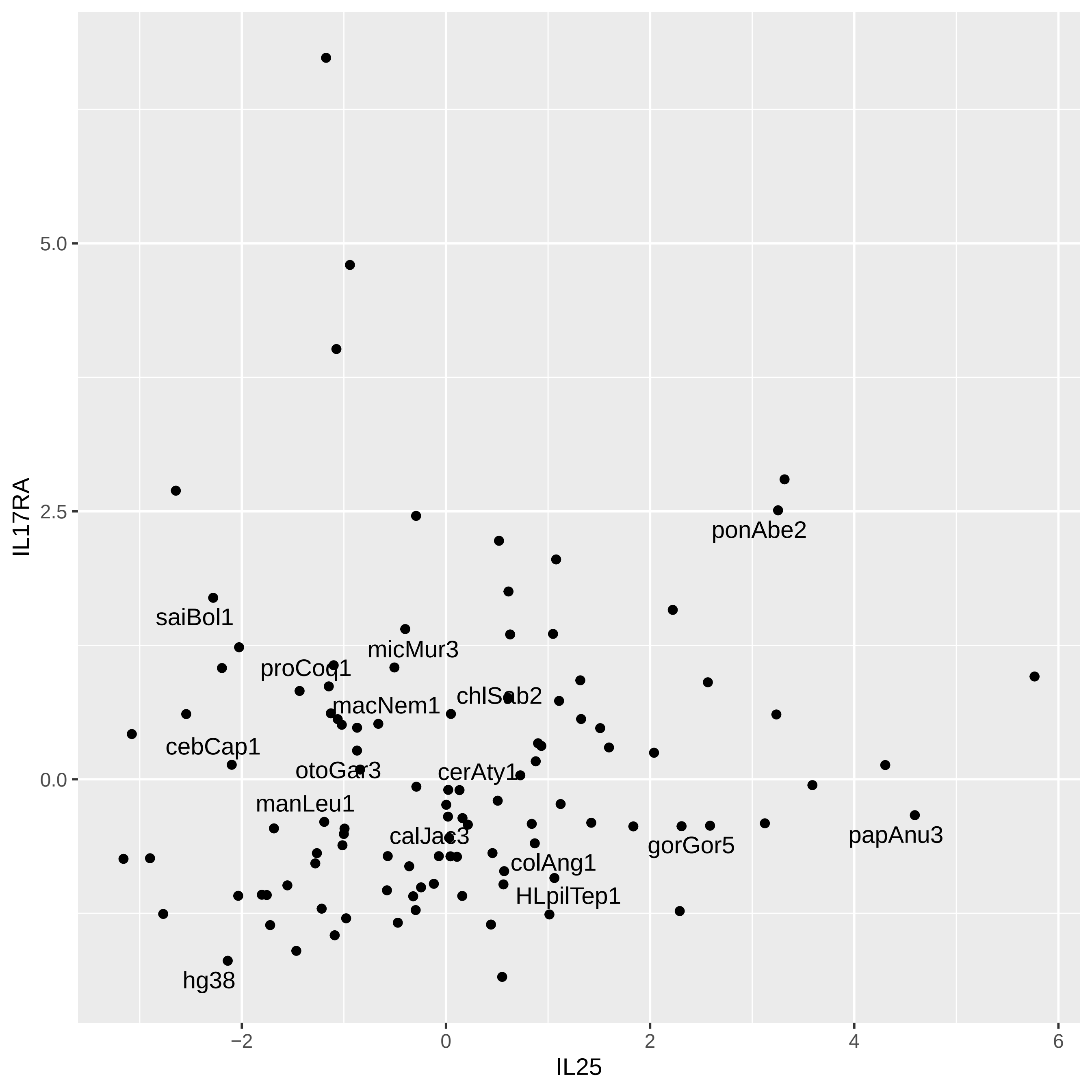
IL17RA
IL25
5.0
2.5
0.0
−2
0
2
4
6
ponAbe2
saiBol1
micMur3
proCoq1
chlSab2
macNem1
cebCap1
otoGar3
cerAty1
manLeu1
calJac3
gorGor5
papAnu3
colAng1
HLpilTep1
hg38
